# Knowledge of HIV Serodiscordance, Transmission, and Prevention among Couples in Durban, South Africa

**DOI:** 10.1371/journal.pone.0124548

**Published:** 2015-04-20

**Authors:** William Kilembe, Kristin M. Wall, Mammekwa Mokgoro, Annie Mwaanga, Elisabeth Dissen, Miriam Kamusoko, Hilda Phiri, Jean Sakulanda, Jonathan Davitte, Tarylee Reddy, Mark Brockman, Thumbi Ndung’u, Susan Allen

**Affiliations:** 1 Rwanda Zambia HIV Research Group, Department of Pathology & Laboratory Medicine, School of Medicine and Hubert Department of Global Health, Rollins School of Public Health, Emory University, Atlanta, Georgia, United States of America; 2 Department of Epidemiology, Rollins School of Public Health, Laney Graduate School, Emory University, Atlanta, Georgia, United States of America; 3 HIV Pathogenesis Programme, Doris Duke Medical Research Institute, University of KwaZulu-Natal, Durban, South Africa; 4 Medical Research Council, Biostatistics Unit, Durban, South Africa; 5 Faculty of Health Sciences and Faculty of Science, Department of Molecular Biology and Biochemistry, Simon Fraser University, Burnaby, British Columbia, Canada; 6 KwaZulu-Natal Research Institute for Tuberculosis and HIV (K-RITH), University of KwaZulu-Natal, Durban, South Africa; University of Maryland School of Medicine, UNITED STATES

## Abstract

**Objective:**

Couples’ voluntary HIV counseling and testing (CVCT) significantly decreases HIV transmission within couples, the largest risk group in sub-Saharan Africa, but it is not currently offered in most HIV testing facilities. To roll out such an intervention, understanding locale-specific knowledge barriers is critical. In this study, we measured knowledge of HIV serodiscordance, transmission, and prevention before and after receipt of CVCT services in Durban.

**Design:**

Pre- and post-CVCT knowledge surveys were administered to a selection of individuals seeking CVCT services.

**Methods:**

Changes in knowledge scores were assessed with McNemar Chi-square tests for balanced data and generalized estimating equation methods for unbalanced data.

**Results:**

The survey included 317 heterosexual black couples (634 individuals) who were primarily Zulu (87%), unemployed (47%), and had at least a secondary level education (78%). 28% of couples proved to be discordant. Only 30% of individuals thought serodiscordance between couples was possible pre‐CVCT compared to 95% post-CVCT. One-third thought there was at least one benefit of CVCT pre‐CVCT, increasing to 96% post‐CVCT. Overall, there were positive changes in knowledge about HIV transmission and prevention. However, many respondents thought all HIV positive mothers give birth to babies with AIDS (64% pre-CVCT, 59% post-CVCT) and that male circumcision does not protect negative men against HIV (70% pre-CVCT, 67% post-CVCT).

**Conclusions:**

CVCT was well received and was followed by improvements in understanding of discordance, the benefits of joint testing, and HIV transmission. Country-level health messaging would benefit from targeting gaps in knowledge about serodiscordance, vertical transmission, and male circumcision.

## Introduction

Poor knowledge of HIV serodiscordance, transmission, and prevention contribute to HIV transmission risk. Knowledge in these domains is low in many sub-Saharan African countries [1–4]. The World Health Organization and UNAIDS highlight that knowledge of HIV/AIDS among women may be especially low [5]. In South Africa, HIV and AIDS knowledge has been the focus of country-wide awareness media campaigns with mixed results [6, 7]. Despite the increased coverage of these campaigns, knowledge of HIV/AIDS, especially regarding prevention, remains poor [8]. Furthermore, poor knowledge in these domains contributes to low prevalence of HIV testing. A population-based survey of men and women in South Africa showed that knowledge of serodiscordance and knowledge of HIV risk behaviors positively predicted HIV testing among HIV-negative persons, and knowledge of the family-level impact of HIV predicted testing among HIV-positive persons [9].

Couples’ voluntary HIV counseling and testing (CVCT) significantly reduces HIV transmission in one of the highest risk groups in sub-Saharan Africa – discordant couples – and has been shown to increase couple’s knowledge about HIV serodiscordance and prevention behaviors [10–19]. Given the high prevalence of HIV among those aged 15–59 (17.9%, 95%CI: 17.3–18.4%) [20] and high prevalence of couple serodiscordance, estimated to be at 27.4% in one study [21], CVCT could be a highly effective HIV prevention intervention in South Africa. Though CVCT is an evidence-based, well studied intervention that has been shown to increase knowledge and decrease HIV transmission between couples, it is not currently offered in most testing facilities in South Africa. To roll out such an intervention, understanding the locale-specific knowledge barriers and ability of CVCT to improve knowledge is critical. In this study, we sought to measure couples knowledge of HIV serodiscordance, transmission, and prevention before and after receipt of CVCT services.

## Methods

### Participant recruitment

The Rwanda Zambia HIV Research Group (RZHRG) pilot tested weekend CVCT services in five clinics in Durban, South Africa as described elsewhere (reference companion paper). Briefly, in collaboration with the HIV Pathogenesis Programme (HPP) at the University of KwaZulu-Natal (UKZN), RZHRG provided support, training, and technical assistance for local Durban counselors and community promoters to implement and pilot test the CVCT intervention from February to June of 2013. CVCT services consist of group pre-test counseling, rapid HIV testing, and joint post-test couple counseling. Client-level indicators (age, gender, pregnancy status, antiretroviral treatment (ART) status, and HIV serostatus) are collected for all couples as a component of routine CVCT service operation. RZHRG CVCT promotions, recruitment [22–24], enrollment, retention [25], testing, and counseling [26, 27] procedures have been described previously.

RZHRG counselor trainers selected participants using simple random sampling from a total of 907 couples attending pilot CVCT services. The target for a cross-sectional survey was at least 300 couples. Participants were consented as a couple and surveyed individually prior to their joint participation in pre-test counseling. Following completion of the pre-test survey, the couple continued through the normal CVCT process together. After receiving their HIV results and completing post-test counseling, the couple was again separated and re-surveyed using the original questionnaire to assess changes in knowledge of HIV transmission, serodiscordance, and HIV prevention measures.

### Survey measures

Survey measures included demographics as well as knowledge and beliefs about serodiscordance and CVCT, HIV transmission, and HIV prevention. Demographic measures included participant age, tribe, religion, occupation, education, years cohabiting, HIV testing history, and history of serostatus disclosure. Client knowledge of the possibility of couple serodiscordance (i.e., knowing that one partner in a couple can be HIV positive while the partner is HIV negative) and perceptions of benefits or disadvantages of CVCT were also ascertained. Participant knowledge of HIV transmission methods (e.g., sex and vertical transmission), misconceptions about transmission (e.g., kissing, mosquito bites, coughing and sneezing), HIV prevention methods (e.g., CVCT, female condoms, male circumcision, prevention of mother-to-child transmission, ART), and misconceptions about prevention (e.g., existence of a vaccine, use of Vaseline or oils with condoms) were assessed using true, false, or don’t know response options.

### Data analysis

Participant demographics were analyzed at the individual level with descriptive statistics (means and standard deviations for continuous variables; counts and frequencies for categorical variables). All variables were stratified by gender. Differences in demographics by gender were evaluated with Chi-square (or Fisher’s exact) tests or t-tests as appropriate.

Participant knowledge scores about serodiscordance and CVCT, captured as categorical variables, were tabulated with descriptive statistics (counts and frequencies) after grouping the “don’t know” response option with the incorrect answer. These response distributions were stratified as pre-CVCT or post-CVCT. Three survey questions pertaining to serodiscordance and CVCT were asked of all participants (i.e., knowledge of serodiscordance, belief that there are benefits to CVCT, and belief that there were disadvantages to CVCT). If a participant answered that serodiscordance was possible, they were then asked about some likely explanations for serodiscordance; similarly, if a participant answered that there were benefits or disadvantages to CVCT, they were then asked about what some of those benefits or disadvantages might be. Thus, these questions were asked conditional upon previous responses and were therefore not balanced between pre- and post-test surveys. To analyze differences in pre- and post-test responses for this unbalanced data, we modeled the response outcome as a function of the pre- or post-test survey and controlling for the initial conditional question using generalized estimating equations (GEE) methods for repeated measures, adjusting for correlation and using the robust covariance. The GEE parameter estimate Z test p-value was compared with p-values obtained from a correlated pre-post analysis (which incorrectly assumes our data is missing at random) and chi-square tests (which incorrectly assume independence between pre- and post-test observations).The interpretations of significant findings did not change regardless of which analysis technique we used.

Participant knowledge scores about transmission and prevention methods and misconceptions, captured as categorical variables, were tabulated with descriptive statistics (counts and frequencies) after grouping the “don’t know” response option with the incorrect answer. Differences in these pre- and post-test scores were quantified using McNemar’s chi-square tests.

We also explored changes in knowledge scores based on couple HIV serostatus that would be of particular relevance (for example, did knowledge of serodiscordance increase among serodiscordant couples? Did knowledge of vertical transmission increase among couples with HIV+ pregnant women?). Finally, we explored whether there were differences in demographic characteristics and serostatus distribution between the sample of CVCT clients who participated in the knowledge survey versus the larger source population of clients receiving CVCT services during the pilot. All analyses were conducted with SAS v9.4 (Cary, NC) and all p-values were two tailed.

### Ethics statement

The study was approved by the Biomedical Research Ethics Committee of the University of KwaZulu-Natal. An ethics committee-approved informed consent form was read to the couple prior to CVCT. The couple was given an opportunity to ask questions, and the form signed by both partners as an indication of voluntary agreement to participate in the knowledge and perception survey.

## Results

### Demographics (Table 1)

The survey included 317 black South African couples (634 individuals) who were primarily of Zulu tribe (87%), Christian (85%), unemployed (47%) or engaged in unskilled manual labor (17%), and had at least a secondary level education (78%). Men were 31 and women were 29 years of age on average. Most participants were not cohabiting with their partner (69%), 62% of participants had previously tested for HIV and of those, 86% reported disclosure to their sexual partner. Most couples (96%) had not participated in CVCT before and did not know that a couple could receive CVCT services in Durban (65%). Women were significantly younger on average (p<0.001), were more likely to be unemployed (55% versus 39%, p<0.001) and Christian (90% versus 81%, P = 0.003), and were more likely to have ever tested for HIV (66% versus 57%, p = 0.02) than men. Among those reporting previous HIV testing, women had tested longer ago than men (24 months versus 20 months, p = 0.27).

**Table 1:** Demographics of clients participating in CVCT and completing pre- and post-CVCT surveys stratified by gender, Durban, South Africa, 2013.

	Total		Men		Women		p-value (2-tailed)
	(N = 634 individuals)		(N = 317)		(N = 317)		
	N	%	N	%	N	%	
**Age (mean, SD)**	29.9	7.2	31.3	7.2	28.6	7.1	<0.0001
**Tribe**							0.83
English	2	0%	1	0%	1	0%	
Siswati	1	0%	1	0%	0	0%	
Sotho	7	1%	3	1%	4	1%	
Xhosa	72	11%	34	11%	38	12%	
Zulu	546	87%	277	88%	269	86%	
**Religion**							0.003
Christian	535	85%	255	81%	280	90%	
None	38	6%	29	9%	9	3%	
Traditional	37	6%	23	7%	14	5%	
Hindu, Muslim, Other	16	3%	9	3%	8	3%	
**Occupation**							<0.0001
Domestic worker	55	9%	12	4%	43	14%	
Professional, Technical, Managerial	14	2%	9	3%	5	2%	
Sales/Services	29	5%	18	6%	11	4%	
Skilled Manual	59	9%	47	15%	12	4%	
Unemployed	294	47%	123	39%	171	55%	
Unskilled manual	107	17%	63	20%	44	14%	
Voluntary work	68	11%	42	13%	26	8%	
**Highest level of education**							0.50
None	18	3%	10	3%	8	3%	
Primary	121	19%	61	19%	60	19%	
Secondary	424	68%	207	66%	217	70%	
Tertiary	63	10%	37	12%	26	8%	
**Years cohabiting**							1.000
Not cohabiting	420	69%	210	69%	210	69%	
≤2 years	102	17%	51	17%	51	17%	
>2 years	90	15%	45	15%	45	15%	
**Have you ever tested for HIV**							0.02
Yes	386	62%	181	57%	205	66%	
No	239	38%	135	43%	104	34%	
**If ever tested, how many days ago? (mean, SD)**	667.1	573.3	614.7	471.3	714.8	650.1	0.03
**If ever tested, did you disclose your HIV status to your partner?**							0.48
Yes	322	86%	158	88%	174	85%	
No	52	14%	22	12%	30	15%	
**Have you and your partner ever tested for HIV together**							0.60
Yes	17	4%	7	3%	10	5%	
No	405	96%	194	97%	211	95%	
**Can a couple receive HIV counseling and testing together in Durban?**							0.26
Yes	195	35%	92	33%	103	37%	
No	360	65%	188	67%	172	63%	

SD: standard deviation.

### Client knowledge and beliefs about serodiscordance and CVCT (Table 2)

There was a low level of knowledge about the possibility of HIV serodiscordance: only 31% thought this was possible pre‐CVCT compared to 95% post-CVCT (p<0.001). Knowledge about possible reasons for discordance (concurrent or previous partners, the negative partner being in the window period of seroconversion, use of condoms or abstinence) was high among those 31% knowing discordance was possible pre-CVCT and significantly (p<0.001) increased post-CVCT (95% of the sample answered those questions). One-third of participants thought there was at least one benefit of CVCT pre‐CVCT; this increased to 96% post‐CVCT. Benefits included facilitated disclosure, planning for the future and risk reduction, all of which were cited by >98% after CVCT.

**Table 2:** Client knowledge and beliefs about serodiscordance and CVCT pre- and post-CVCT, Durban, South Africa, 2013.

	Pre-test responses		All post-test responses		p-value (2-tailed)*
	N	%	N	%	
**When a couple undergoes CVCT, can their HIV results be different (knowledge of discordance)?**					
Yes	193	31%	578	95%	<.001
No	430	69%	32	5%	
**If discordance is possible, what are the likely explanations?**					
**HIV+ partner could have gotten HIV from another source and has not transmitted to the negative partner**					
Prompted/Spontaneous	148	95%	563	99%	0.01
No	7	5%	7	1%	
**HIV negative partner could be in the window period**					
Prompted/Spontaneous	148	89%	567	99%	<.001
No	18	11%	6	1%	
**The couple has been using condoms**					
Prompted/Spontaneous	151	93%	568	98%	<.001
No	11	7%	9	2%	
**The couple has been abstaining**					
Prompted/Spontaneous	140	87%	543	96%	<.001
No	21	13%	20	4%	
**Are there any benefits to CVCT?**					
Yes	206	33%	585	96%	<.001
No	411	67%	24	4%	
**If yes there are benefits, what are some benefits?**					
**Individuals are not burdened with disclosing their HIV status to their partner**					
Prompted/Spontaneous	175	96%	570	99%	<.01
No	8	4%	6	1%	
**The couple can plan for their future together**					
Prompted/Spontaneous	182	94%	574	99%	<.01
No	11	6%	7	1%	
**Counseling facilitates mutual cooperation for risk reduction**					
Prompted/Spontaneous	177	93%	572	98%	<.01
No	13	7%	9	2%	
**Are there any disadvantages of undergoing CVCT?**					
Yes	28	5%	28	5%	1.00
No	583	95%	583	95%	

*GEE parameter estimate Z test p-value.

At baseline, 5% of all participants thought there were disadvantages to participating in CVCT, and this did not decrease post-CVCT. However, the main disadvantage stated (fear of relationship dissolution) decreased significantly pre- to post-service (54% pre-CVCT, which fell to 30% post-CVCT, data not shown, p<0.05).

### Client knowledge and beliefs about HIV transmission (Table 3)

Client knowledge scores related to sexual HIV transmission were generally high pre-CVCT. Most improved significantly (p<0.01) pre- to post-test. Some exceptions included the proportion who believed that a woman could not get HIV if she had sex during her period (39% pre-CVCT) which improved somewhat though persisted in a substantial minority after CVCT (30% post-CVCT). Similarly, many people thought and continued to think that all HIV positive mothers give birth to babies with AIDS (64% pre-CVCT and 59% post-CVCT). The proportion of respondents who believed that a person could get HIV by deep kissing a partner with HIV remained stable at 31%-32%. Misconceptions about casual transmission (sharing a tub, mosquito bites, coughing/sneezing) were uncommon pre-CVCT and were further reduced after CVCT.

**Table 3:** Client knowledge and beliefs about HIV transmission and prevention pre- and post-CVCT, Durban, South Africa, 2013.

	Pre-test responses		Post-test responses		McNemar's chi-square p-value (2-tailed)
	N	%	N	%	
**Knowledge and beliefs about HIV transmission**					
**Having sex with more than one partner can increase a person’s chance of being infected with HIV**					<.001
True	583	95%	604	99%	
Don't know/False	28	5%	7	1%	
**A woman CANNOT get HIV if she has sex during her period**					<.0001
False	374	61%	429	70%	
Don't know/True	240	39%	185	30%	
**All pregnant women infected with HIV will have babies born with AIDS**					0.001
False	222	36%	255	41%	
Don't know/True	393	64%	360	59%	
**People are likely to get HIV by deep kissing if their partner has HIV**					0.140
False	201	32%	189	31%	
Don't know/True	418	68%	430	69%	
**A person can get HIV by sitting in a hot tub or a swimming pool with another person who has HIV**					<.0001
False	551	89%	594	96%	
Don't know/True	68	11%	25	4%	
**HIV can be transmitted by a mosquito bite**					<.0001
False	511	83%	580	94%	
Don't know/True	106	17%	37	6%	
**Coughing and sneezing DO NOT spread HIV**					<.0001
True	520	84%	573	93%	
Don't know/False	99	16%	46	7%	
**Knowledge and beliefs about HIV prevention**					
**Testing for HIV with one’s sex partner helps to reduce HIV transmission**					0.30
True	575	94%	582	95%	
Don't know/False	38	6%	31	5%	
**HIV medicine provided to an HIV pregnant woman can reduce the chances of HIV transmission to her baby**					0.01
True	573	93%	592	96%	
Don't know/False	43	7%	24	4%	
**There is a female condom that can help decrease a woman’s chance of getting HIV**					0.001
True	550	90%	576	94%	
Don't know/False	60	10%	34	6%	
**Male circumcision DOES NOT protect HIV negative men from acquiring HIV**					0.03
False	180	30%	203	33%	
Don't know/True	426	70%	403	67%	
**Use of HIV medication in an infected person can reduce the chances of transmitting HIV to his or her partner**					0.03
True	369	61%	390	64%	
Don't know/False	237	39%	216	36%	
**There is a vaccine that can stop adults from getting HIV**					0.10
False	361	60%	384	64%	
Don't know/True	242	40%	219	36%	
**Using Vaseline or baby oil with condoms lowers the chance of getting HIV**					<.001
False	446	72%	520	84%	
Don't know/True	172	28%	98	16%	

### Client knowledge and beliefs about HIV prevention (Table 3)

Knowledge with regards to HIV prevention increased pre- to post-CVCT, though these increases were not always statistically significant. Pre- and post-CVCT, most people thought that testing with one’s partner helps reduce HIV transmission (94% and 95%, respectively). The proportion of people who knew that medicine can be provided to prevent perinatal transmission and that female condoms can prevent HIV were high pre-CVCT (90–93%) and both increased significantly post-CVCT (94–96%). However, many people thought that male circumcision does not protect HIV negative men against HIV (70% pre-CVCT and 67% post-CVCT), that use of HIV medication in an infected person does not reduce chances of transmission (39% pre-CVCT and 36% post-CVCT), that there is a vaccine that can prevent adults from getting HIV (40% pre-CVCT and 36% post-CVCT), and that use of Vaseline or baby oil with condoms lowers the chance of getting HIV (28% pre-CVCT and 16% post-CVCT).

### Serostatus-specific differences in knowledge scores (data not shown)

Among the n = 88 HIV discordant couples, 80% of both men and women did not think that serodiscordance was possible pre-CVCT. 3% of both men and women still did not think that a couples’ HIV test results can be different at post-test (i.e., after receiving their own discordant test results). Among the n = 15 couples with HIV+ pregnant women, knowledge that HIV infected women do not always have babies born with HIV did not change significantly pre-CVCT (22%) to post-CVCT (25%).

### Comparison of the knowledge survey sample and all couples tested

The subset of CVCT clients who were randomly selected and who participated in the knowledge survey are similar to the source population of all couples receiving CVCT on demographics (age, cohabitation status, previous HIV testing, and serostatus distribution). Couples answering the knowledge survey were not significantly more likely to have been previously tested individually (66%) versus couples from the source population (63%).

## Discussion

In this group of couples who sought CVCT services, the majority of whom had not been previously tested and counseled together, CVCT resulted in improved knowledge about the possibility of discordant results, the benefits of CVCT, and mechanisms of transmission and prevention. Knowledge gaps remained in areas that were not a specific focus of CVCT, including prevention of mother-to-child transmission and male circumcision.

The majority (69%) of individuals did not know discordance was possible before receiving CVCT services. Understanding that serodiscordance between couples is possible and a risk factor for transmission is key to HIV prevention. Recent findings from a qualitative study of 50 serodiscordant couples seeking HIV services in Durban found that misunderstanding serodiscordance was related to HIV risk behavior [1]. Additionally, low knowledge of the possibility of couple serodiscordance has also been shown to be a barrier to CVCT uptake [3]. Given the extremely high prevalence of couple serodiscordance in the region (estimated at 28.0% is this survey, 29.5% in our larger pilot study (reference companion paper) and 27.4% in the Partners in Prevention HSV-2/HIV-1 Transmission study [21]), country-level health messaging would benefit from targeting baseline gaps in knowledge about serodiscordance to increase testing and decrease transmission. Interestingly, our serostatus-specific analyses showed that inaccurate knowledge about serodiscordance persists at a very low level (3%) among discordant couples even after receiving their discordant test results, indicating either active disbelief or a persistent lack of understanding of the concept of serodiscordance.

At baseline, very few (5%) participants thought there were disadvantages to participating in CVCT, and this did not change post-CVCT. Stated disadvantages largely concerned relationship dissolution (54% pre-CVCT, which fell to 30% post-CVCT). Recently published results from formative in-depth interviews with 10 couples indicated that suspicion of infidelity was a main barrier for young South African couples to engage in couples-based HIV prevention services and discussions [28]. Another recently-published study from South Africa that evaluated home-based CVCT outcomes indicated that positive status was seen as an indicator of infidelity that could negatively affect relationship trust [29]. Though not specifically found here, suspicions of infidelity and relationship trust are important issues to address during pre-and post-test counseling and are part of standard counselor training.

We also found that though most misconceptions about transmission and prevention were uncommon, there was a common and persistent belief that all HIV positive pregnant women will give birth to a HIV positive child (64% pre-CVCT and 59% post-CVCT). In our serostatus-specific analysis, accurate knowledge of mother-to-child transmission remained low among pregnant couples in which the woman was HIV positive. We also found that knowledge of the protective effect of male circumcision was low and persisted after CVCT. Poor understanding of mother-to-child transmission and male circumcision merit more investigation. For example, it may be that respondents thought mother-to-child transmission WITHOUT ARV would always result in transmission because the question did not specify ARV status. Similarly, respondents may have heard that male circumcision reduces risk but understood that if a circumcised man has unprotected sex he could still get HIV, which is correct. A clearer picture of these subtleties would help craft accurate and helpful country-level health messaging. Finally, misconceptions about topics that are not explicitly addressed in the CVCT materials, such as reduced risk via use of Vaseline or oil with male condoms and the existence of an HIV vaccine, were reported. These misconceptions may come up during group, pre-, or post-test counseling sessions, and counselors must be prepared to address these on a case-by-case basis.

Though there has been debate about whether CVCT will be an acceptable and effective intervention in South Africa due to the lower prevalence of marriage/co-habitation and potentially higher rates of multiple concurrent partners reported in that country, recent studies suggest concurrent partnerships are not driving high HIV incidence in this area [30]. Additionally, our findings show that, though most participants were not cohabiting (69%), the acceptability of CVCT was still very high for couples of any cohabitation status, with the majority (96%) of participants reporting benefits of CVCT after receiving the service, such as planning for the future and risk reduction.

Despite the World Health Organization’s endorsement of CVCT as an HIV prevention strategy [12], neither the US President’s Emergency Plan for AIDS Relief (PEPFAR) [31] nor the Global Fund [32] have historically included couples’ testing among their required indicators nor has any significant funding been directed at the inclusion of partners in prevention of mother-to-child transmission, ART, individual HIV counseling and testing, or male circumcision programs. It is not clear why CVCT receives so little support. Our experience implementing CVCT has shown that initial training costs are modest and implementation saves time as a couple can be tested and counseled more efficiently than two individuals. Active promotions are a small fraction of program costs but are essential to dispel the misconception that couples must share the same HIV status. Coercion is a theoretical concern and counselor training materials include strategies to avoid this [33]. Fortunately, violence has not been a feature of large CVCT programs in Rwanda (where CVCT has been nationalized in antenatal clinics) nor in Zambia (where the training team for this Durban program have hosted more than 150,000 couples in government clinics).

Our findings should be interpreted after consideration of the potential for information bias that could occur if questions were not understood or were misinterpreted. This bias may be differential by educational level. Additionally, couples who participated in the survey are not reflective of the general population, but have self-selected to access CVCT services. There were no significant differences between those selected for the survey and the larger pilot study population in terms of age, cohabitation, previous HIV testing, and couple serostatus (data not shown), and we believe this survey sample represents that larger population. However, the sample and pilot study populations could have differed by factors that we did not measure in the larger pilot study, such as education. Finally, we have not yet measured the longitudinal impact of the knowledge gained or evaluated whether the increased knowledge scores translated into longer-term increases in risk reduction.

Despite these limitations, the majority of respondents had a positive attitude towards CVCT and viewed the service as beneficial. Importantly, CVCT was able to provide tailored counseling based on the combination of both partners’ test results – particularly important for the many discordant couples that did not previously know discordance was possible – and lessened misconceptions in most domains. We recommend that both country-level health messaging and CVCT service messaging target locale-specific gaps in knowledge about serodiscordance and the benefits of CVCT, and pay special attention to targeted prevention of mother-to-child transmission and male circumcision messaging.

## References

[1] Matthews LT, Crankshaw T, Giddy J, Kaida A, Smit JA, Ware NC, et al Reproductive decision-making and periconception practices among HIV-positive men and women attending HIV services in Durban, South Africa. AIDS and behavior. 2013;17(2):461–70. 10.1007/s10461-011-0068-y 22038045 PMC3560938

[2] Castle S. Doubting the existence of AIDS: a barrier to voluntary HIV testing and counselling in urban Mali. Health policy and planning. 2003;18(2):146–55. 12740319 10.1093/heapol/czg019

[3] Kelley AL, Karita E, Sullivan PS, Katangulia F, Chomba E, Carael M, et al Knowledge and perceptions of couples' voluntary counseling and testing in urban Rwanda and Zambia: a cross-sectional household survey. PloS one. 2011;6(5):e19573 10.1371/journal.pone.0019573 21573068 PMC3090401

[4] King R, Wamai N, Khana K, Johansson E, Lindkvist P, Bunnell R. "Maybe his blood is still strong": a qualitative study among HIV-sero-discordant couples on ART in rural Uganda. BMC public health. 2012;12:801 10.1186/1471-2458-12-801 22984868 PMC3599002

[5] WHO. Gender inequalities and HIV: WHO; 2014 Available: http://www.who.int/gender/hiv_aids/en/. Accessed 14 June 2014.

[6] Johnson S, Kincaid L, Laurence S, Chikwava F, Delate R, Mahlasela L. Second National HIV Communication Survey 2009 Pretoria: JHHESA; 2010 Available: http://www.uj.ac.za/EN/CorporateServices/ioha/surveys/Documents/ncs_report.pdf. Accessed 22 May 2014.

[7] Salaam-Blyther T. The Global Fund to Fight AIDS, Tuberculosis, and Malaria: Progress Report and Issues for Congress Congressional Research Service, The Library of Congress2006. Available: http://fpc.state.gov/documents/organization/66458.pdf. Accessed 22 May 2014.

[8] Human Sciences Research Council. South African National HIV Prevalence, Incidence, Behaviour and Communication Survey, 2008 Cape Town, South Africa: HSRC Press; 2009 Available: http://www.mrc.ac.za/pressreleases/2009/sanat.pdf. Accessed 22 May 2014.

[9] Peltzer K, Matseke G, Mzolo T, Majaja M. Determinants of knowledge of HIV status in South Africa: results from a population-based HIV survey. BMC public health. 2009;9:174 10.1186/1471-2458-9-174 19500373 PMC2700104

[10] Chemaitelly H, Awad SF, Shelton JD, Abu-Raddad LJ. Sources of HIV incidence among stable couples in sub-Saharan Africa. Journal of the International AIDS Society. 2014;17:18765 10.7448/IAS.17.1.18765 24560339 PMC3935448

[11] Dunkle KL, Stephenson R, Karita E, Chomba E, Kayitenkore K, Vwalika C, et al New heterosexually transmitted HIV infections in married or cohabiting couples in urban Zambia and Rwanda: an analysis of survey and clinical data. Lancet. 2008;371(9631):2183–91. 10.1016/S0140-6736(08)60953-8 18586173

[12] World Health Organization Guidance on couples HIV testing and counselling—including antiretroviral therapy for treatment and prevention in serodiscordant couples Geneva, Switzerland: WHO Press; 2012 Available: http://apps.who.int/iris/bitstream/10665/44646/1/9789241501972_eng.pdf?ua=1. Accessed 26 February 2014. 23700649

[13] Allen S, Tice J, Van de Perre P, Serufilira A, Hudes E, Nsengumuremyi F, et al Effect of serotesting with counselling on condom use and seroconversion among HIV discordant couples in Africa. BMJ. 1992;304(6842):1605–9. 1628088 10.1136/bmj.304.6842.1605 PMC1881972

[14] Fideli US, Allen SA, Musonda R, Trask S, Hahn BH, Weiss H, et al Virologic and immunologic determinants of heterosexual transmission of human immunodeficiency virus type 1 in Africa. AIDS research and human retroviruses. 2001;17(10):901–10. 11461676 10.1089/088922201750290023 PMC2748905

[15] Guthrie BL, de Bruyn G, Farquhar C. HIV-1-discordant couples in sub-Saharan Africa: explanations and implications for high rates of discordancy. Current HIV research. 2007;5(4):416–29. 17627505 10.2174/157016207781023992

[16] Hira SK, Nkowane BM, Kamanga J, Wadhawan D, Kavindele D, Macuacua R, et al Epidemiology of human immunodeficiency virus in families in Lusaka, Zambia. Journal of acquired immune deficiency syndromes. 1990;3(1):83–6. 2293646

[17] Kamenga M, Ryder RW, Jingu M, Mbuyi N, Mbu L, Behets F, et al Evidence of marked sexual behavior change associated with low HIV-1 seroconversion in 149 married couples with discordant HIV-1 serostatus: experience at an HIV counselling center in Zaire. AIDS. 1991;5(1):61–7. 2059362 10.1097/00002030-199101000-00009

[18] Becker S, Mlay R, Schwandt HM, Lyamuya E. Comparing couples' and individual voluntary counseling and testing for HIV at antenatal clinics in Tanzania: a randomized trial. AIDS and behavior. 2010;14(3):558–66. 10.1007/s10461-009-9607-1 19763813

[19] Allen S, Meinzen-Derr J, Kautzman M, Zulu I, Trask S, Fideli U, et al Sexual behavior of HIV discordant couples after HIV counseling and testing. AIDS. 2003;17(5):733–40. 12646797 10.1097/00002030-200303280-00012

[20] UNAIDS. HIV and AIDS estimates 2012. Available: http://www.unaids.org/en/regionscountries/countries/southafrica/. Accessed 12 May 2014.

[21] Lingappa JR, Lambdin B, Bukusi EA, Ngure K, Kavuma L, Inambao M, et al Regional differences in prevalence of HIV-1 discordance in Africa and enrollment of HIV-1 discordant couples into an HIV-1 prevention trial. PLOS one. 2008;3(1):e1411 10.1371/journal.pone.0001411 18183292 PMC2156103

[22] Wall KM, Kilembe W, Nizam A, Vwalika C, Kautzman M, Chomba E, et al Promotion of couples' voluntary HIV counselling and testing in Lusaka, Zambia by influence network leaders and agents. BMJ open. 2012;2(5). 10.1136/bmjopen-2012-001171 PMC3467632 22956641

[23] Wall K, Karita E, Nizam A, Bekan B, Sardar G, Casanova D, et al Influence network effectiveness in promoting couples’ HIV voluntary counseling and testing in Kigali, Rwanda. AIDS. 2012;26(2):217–27. 10.1097/QAD.0b013e32834dc593 22008653 PMC3679893

[24] Allen S, Karita E, Chomba E, Roth DL, Telfair J, Zulu I, et al Promotion of couples' voluntary counselling and testing for HIV through influential networks in two African capital cities. BMC public health. 2007;7:349 18072974 10.1186/1471-2458-7-349 PMC2241615

[25] Kempf MC, Allen S, Zulu I, Kancheya N, Stephenson R, Brill I, et al Enrollment and retention of HIV discordant couples in Lusaka, Zambia. Journal of acquired immune deficiency syndromes. 2008;47(1):116–25. 18030162 10.1097/QAI.0b013e31815d2f3f

[26] Chomba E, Allen S, Kanweka W, Tichacek A, Cox G, Shutes E, et al Evolution of couples' voluntary counseling and testing for HIV in Lusaka, Zambia. Journal of acquired immune deficiency syndromes. 2008;47(1):108–15. 17984761 10.1097/QAI.0b013e31815b2d67

[27] Boeras DI, Luisi N, Karita E, McKinney S, Sharkey T, Keeling M, et al Indeterminate and discrepant rapid HIV test results in couples' HIV testing and counselling centres in Africa. Journal of the International AIDS Society. 2011;14:18 10.1186/1758-2652-14-18 21477317 PMC3086828

[28] Parker L, Pettifor A, Maman S, Sibeko J, Macphail C. Concerns about partner infidelity are a barrier to adoption of HIV-prevention strategies among young South African couples. Culture, health & sexuality. 2014:1–14. 10.1080/13691058.2014.905707 PMC4832568 24816215

[29] Tabana H, Doherty T, Rubenson B, Jackson D, Ekstrom AM, Thorson A. 'Testing Together Challenges the Relationship': Consequences of HIV Testing as a Couple in a High HIV Prevalence Setting in Rural South Africa. PLOS ONE. 2013;8(6):e66390 23824067 10.1371/journal.pone.0066390 PMC3688905

[30] Tanser F, Barnighausen T, Hund L, Garnett GP, McGrath N, Newell ML. Effect of concurrent sexual partnerships on rate of new HIV infections in a high-prevalence, rural South African population: a cohort study. Lancet. 2011;378(9787):247–55. 10.1016/S0140-6736(11)60779-4 21763937 PMC3141142

[31] Relief TPsEPfA. Next Generation Indicators Reference Guide 2013. Available: http://www.pepfar.gov/documents/organization/206097.pdf. Accessed 02 February 2015.

[32] The Global Fund to Fight AIDS TaM. Core List of Indicators 2014. Available: http://www.theglobalfund.org/en/me/documents/indicatorslist/. Accessed 02 February 2015.

[33] CDC. Couples HIV Counseling and Testing Intervention and Training Curriculum Atlanta, GA: Centers for Disease Control and Prevention; 2011. Available from: cdc.gov/globalaids/Resources/prevention/chct.html. Accessed 27 June 2014.

